# Ivabradine–Flecainide as Breakthrough Drug Combination for Congenital Junctional Ectopic Tachycardia: A Case Report and Literature Review

**DOI:** 10.3390/pediatric13040074

**Published:** 2021-11-23

**Authors:** Giovanni Maria Di Marco, Angelica De Nigris, Angela Pepe, Annamaria Pagano, Giangiacomo Di Nardo, Vincenzo Tipo

**Affiliations:** 1 Division of Cardiology, Department of Pediatrics, Santobono- Pausilipon Children Medical Hospital, 80129 Naples, Italy; giovanni.m.dimarco@gmail.com (G.M.D.M.); gg.dinardo@libero.it (G.D.N.); 2 Department of Woman, Child and General and Specialist Surgery, University of Campania “Luigi Vanvitelli”, 80138 Naples, Italy; 3 Department of Medicine, Surgery and Dentistry “Scuola Medica Salernitana”, Pediatrics Section, University of Salerno, 84081 Baronissi, Italy; angpepe01@gmail.com; 4 Department of Translational Medical Science, Pediatrics Section, University of Naples “Federico II”, 80126 Naples, Italy; annamaria16392@gmail.com; 5 Pediatric Emergency and Short Stay Unit, Santobono-Pausilipon Children’s Hospital, 80129 Naples, Italy; enzotipo@libero.it

**Keywords:** ivabradine, tachycardia, junctional, pediatrics, congenital

## Abstract

Congenital junctional ectopic tachycardia (CJET) is a rare tachyarrhythmia that remains difficult to manage, with suboptimal control in most cases. Here, we report literature research on the use of ivabradine in the treatment of pediatric junctional ectopic tachycardia (JET), both congenital and postoperative, and describe the successful use of ivabradine–flecainide association for CJET therapy resistant to other antiarrhythmic agents. This new drug combination was effective in completely suppressing JET. Ivabradine–flecainide combination may be considered a new therapeutic strategy of CJET with a satisfactory efficacy/tolerability ratio in patients resistant to conventional drug combinations.

## 1. Introduction

Junctional ectopic tachycardia (JET) takes its origin from the atrioventricular (AV) node and AV junction, also including the bundle of the His complex (BH) [1]. Based on the etiology, it is possible to classify JET into congenital and postoperative.

Congenital junctional ectopic tachycardia (CJET) is a rare arrhythmia that can occur in infants with a structurally normal heart and without previous cardiac surgery. This is often refractory to conventional medical therapy. Persistent JET in children can result in ventricular dysfunction, heart failure and high morbidity and mortality [1,2].

Ivabradine is a novel heart-rate-controlling drug that acts by inhibiting the funny current responsible for the spontaneous depolarization of cardiac pacemaker cells.

In this paper, we conduct a literature review on the use of ivabradine in children with JET, both congenital and postoperative.

In addition to this, we describe the therapeutic management of CJET using the new and never described before association of ivabradine with flecainide.

## 2. Case Report

**Figure 1:**
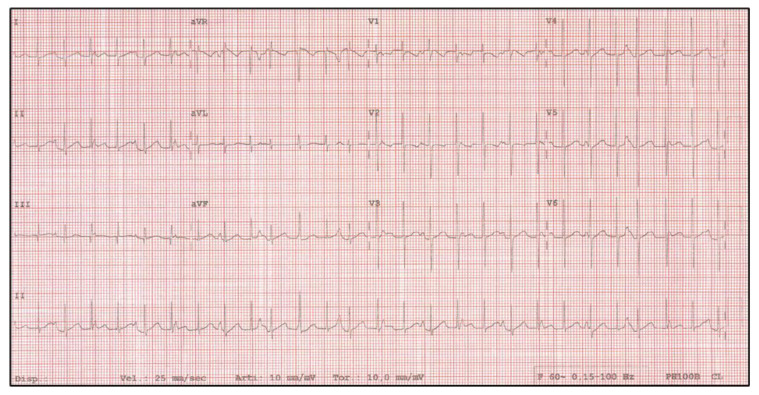
JET at diagnosis.

We report the case of a one-year-old female patient. On day two of life, CJET was diagnosed (Figure 1) in the absence of structural cardiopathies or previous surgery.

The patient had a long hospitalization, during which she was monitored using continuous ECG, several Holter ECG and 12-lead ECG more than once a day, as well as echocardiographic evaluation and periodical biochemistry.

Amiodarone (300 mg/m^2^/d) was started as first-line therapy, leading to satisfactory heart rate control. Later onset of severe hypothyroidism (twenty days after beginning the therapy) induced withdrawal of amiodarone and introduction of flecainide (5 mg/kg/d) and propranolol (4 mg/kg/d). This second-line therapy did not show acceptable arrhythmia control.

For this reason, a third-line therapy was started with propranolol (4 mg/kg/d) in combination with ivabradine (0.25 mg/kg/d). This association was able to ensure satisfactory heart rate control, but without restoring sinus rhythm. Despite this, the patient was discharged with this therapeutic regimen and underwent follow-up.

**Figure 2:**
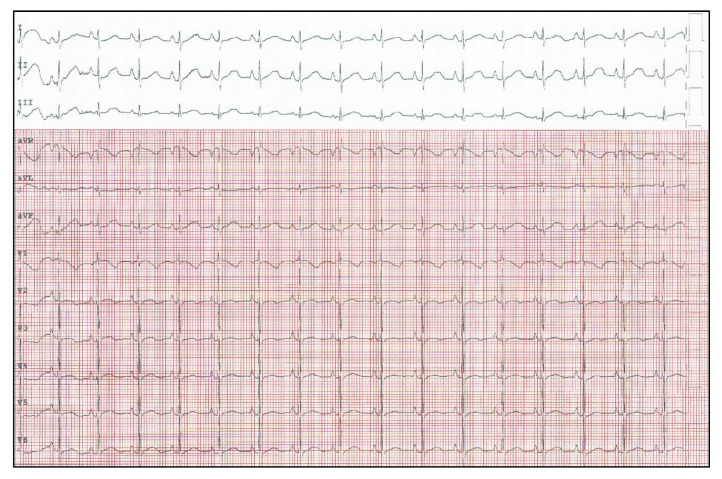
Restoration of sinus rhythm in patient with JET treated with ivabradine in combination with flecainide.

After four months of follow-up, the recurrent elevated heart rate associated with echocardiographic evidence of biatrial and left ventricle dilation led the patient to rehospitalization. In agreement with fellow endocrinologists, amiodarone was reintroduced as the best therapeutic option, although with the risk of hypothyroidism. High doses of amiodarone (350 mg/m^2^/d) alone were not able to prevent high heart rate phases. Therefore, ivabradine (0.25 mg/kg/d) was added with better heart rate control but not as expected. Consequently, it was decided to try a new therapeutic strategy, never tried before in the management of JET. Ivabradine (0.3 mg/kg/d) and flecainide (5 mg/kg/d) association was introduced, resulting in complete conversion to normal sinus rhythm within a few hours (Figure 2).

During sleep, the patient showed a too-low heart rate. For this reason, the doses of both drugs were reduced as follows: ivabradine, 0.25 mg/kg/d; flecainide, 4 mg/kg/d. This therapeutic choice led to satisfactory results, including excellent heart rate values, most of the time in sinus rhythm, with only a few hours per day in junctional rhythm at heart rate values not much higher than those in sinus rhythm.

The patient’s parents were correctly informed about the use of a drug in the off-label regimen, and proper informed consent about potential risks and unexpected drug behaviors was given.

**Figure 3:**
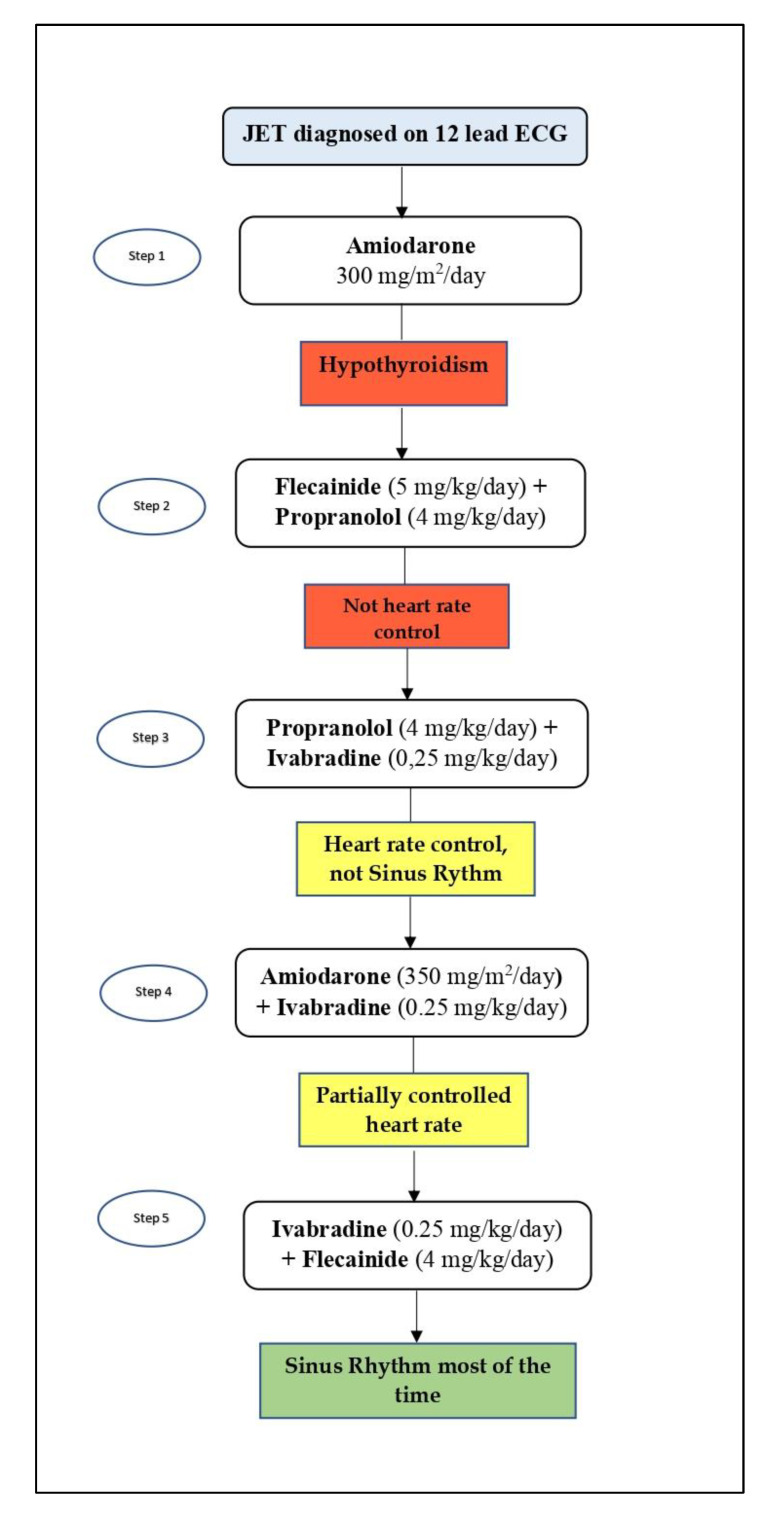
CJET management in our case, step by step.

At discharge, echocardiography showed normal-sized cardiac chambers and normal biventricular function. After four months of follow-up, to date, this therapy continues to show satisfactory results in terms of clinical conditions and control of JET. Provided therapies step by step are summarized in Figure 3.

## 3. Discussion

CJET is a tachyarrhythmia that can appear in infants without structural cardiac abnormalities or previous cardiac surgery. Normal sinus rhythm is characterized by the propagation of an electrical signal originating in the sinus node through the atrium, then through the AV node to the ventricle via bundle branches and the His–Purkinje system. Spontaneous depolarization in JET arises in the AV node and is directed to the ventricle with or without retrograde conduction into the atrium. A postulated mechanism of enhanced automaticity would make clear why adenosine and direct-current cardioversion are ineffective, highlighting the automatic rather than reentrant nature of this tachycardia [1].

Medical management is a challenge for most patients who typically require two or more antiarrhythmics for adequate tachycardia control [1,2].

The first-line recommended therapy for JET is amiodarone. It can be administered intravenously or orally according to the severity of symptoms. If the response to amiodarone is unsatisfactory, the use of digoxin, a beta-blocking agent or flecainide is recommended [3].

Amiodarone toxicity affects the skin, nerves, liver, thyroid gland, eyes and lungs. The frequency of most adverse effects is related to the dosage and duration of treatment. Therefore, it is recommended to use the lowest possible dosage of amiodarone, and, if necessary, the appearance of side effects should lead to the discontinuation of treatment [4].

The reduction of amiodarone dose and its potential long-term toxicity can be obtained by adding a second drug [5,6] such as beta-blockers, digoxin and flecainide [1,2,3,7,8,9].

The combination of flecainide and propranolol has also been described as an effective alternative therapy for CJET [10].

Ivabradine is a heart rate-lowering agent that acts in the sinus node, inhibiting the pacemaker I*_f_* current [11]. It is widely used to decrease sinus rate in the treatment of cardiac failure [12,13]. Recently, it has also been described to successfully treat JET in children with rapid rate control and establishment of sinus rhythm [14,15,16].

This could also be explained by the presence of these pacemaker currents in the AV node and His–Purkinje cells of the cardiac conduction system, as well as in immature ventricular myocardium. I*_f_* current is a sodium current that flows through channels consisting of tetramers of HCN-channel proteins whose predominant isoforms are HCN4 in the sinus node and HCN3 in the AV node [17].

In this paper, we conducted literature research on the use of ivabradine in postoperative and congenital JET. PubMed and Google Scholar databases were used with the following keywords: “Junctional Ectopic Tachycardia”; “children”; “Ivabradine”. We excluded guidelines, reviews, adult studies (>18 years), commentaries, and case series already described in another article. A final set of 11 articles was appropriate for the scope of our review (Table 1).

As shown in Table 1, the use of ivabradine in JET treatment, alone or in combination with other antiarrhythmics, has been described by several authors [14,16,18,19,20,21,22,23,24,25].

Except for some findings of bradycardia, ivabradine is generally well tolerated and, in a great number of cases, has led to good heart rate control and restoration of the sinus rhythm.

When focusing on congenital JET, we found five papers describing the use of ivabradine.

Dieks et al., based on the outcomes observed in five patients, proposed the use of ivabradine associated with other antiarrhythmic agents such as amiodarone in children with CJET [18].

Al-Ghamdi et al. reported a case of CJET in a three-year-old female who switched to sinus rhythm after a second oral dose of ivabradine [14].

Ergul et al. described three infants with treatment-refractory congenital JET despite multiple antiarrhythmics (amiodarone and flecainide combined with either propranolol or digoxin) and quickly converted to sinus rhythm with ivabradine treatment [16]. The authors, however, pointed out that using ivabradine as monotherapy may not be as effective as in combination therapy.

Kothari et al. reported two siblings with CJET unsatisfactory controlled with multiple antiarrhythmic agents, including propranolol, flecainide and amiodarone, and significantly converted to sinus rhythm after the first dose of ivabradine [15].

Rios et al. described cases of two patients affected by congenital JET with inadequate response to other antiarrhythmic medications [19]. Treatment with ivabradine showed a brilliant clinical response.

Although the combination of ivabradine with other antiarrhythmic agents has already been described, ivabradine in association with flecainide alone, as reported here, is indeed new, and this may be the first description of this combination therapy for infants with CJET.

## 4. Conclusions

The present study suggests that the ivabradine–flecainide combination is an alternative effective therapy for CJET, with a satisfactory efficacy/tolerability ratio in patients resistant to conventional antiarrhythmic associations.

Further studies on a larger patient group are imperative to understand the efficacy and safety of this new drug combination.

## Figures and Tables

**Table 1:** Use of ivabradine in pediatric JET: review of the literature.

	Author	Number of Patients (N); Gender	Antiarrhythmic Medication Before Ivabradine	Treatment with Ivabradine (Dose)	Outcome, Response to Ivabradine	Antiarrhythmic Medication with Ivabradine	Adverse Reactions
**Congenital** **JET**	Al-Ghamdi et al. [14]	N = 1; F	Flecainide, sotalol, procainamide, amiodarone	2.5 mg once daily	SR, HR control	None	None
	Dieks et al. [18]	N = 5; 2 M, 3 F	Amiodarone, n = 5 Flecainide, n = 1 Digoxin, n =2	0.05–0.1 mg/kg increased up to 0.28 mg/kg/d	HR control, n = 5 SR, n = 3 JR/JET, n = 1 JR/SR, n = 1	Amiodarone, n = 5 Propranolol, amiodarone, n = 2 Digoxin, amiodarone, flecainide, n = 1	None
	Ergul et al. [16]	N = 3; 2 M, 1 F	Flecainide, amiodarone, digoxin, n = 1 Flecainide, amiodarone, propranolol, n = 2	0.1 mg/kg/d	HR control, n = 3 SR, n = 2 JR/SR, n = 1	Amiodarone, n = 1 Amiodarone, propranolol, flecainide, n = 2	None
	Kothari et al. [15]	N = 2; 1 M, 1 F	Amiodarone, propranolol, flecainide, n = 2	0.5 mg/kg/dose	SR, HR control	Amiodarone, propranolol, flecainide, n = 2	None
	Rios et al. [19]	N = 2; 2 M	Amiodarone, flecainide, propranolol, n = 1 Propranolol, amiodarone, n = 1	0.05/mg/kg/dose	HR control, n = 2 SR/TN, n = 2	Amiodarone, n = 2	None
**Postoperative** **JET**	Khan et al. [20]	N = 7 (6 Jet); 5 M, 2 F	Amiodarone, n = 7	0.05 mg/kg/dose	SR, n = 4 HR control with persistent slow JET, n = 1	Amiodarone	None
	Krishna et al. [21]	N = 8; 4 M, 4 F	Amiodarone, n = 1 Overdrive pacing, n = 5	0.05 mg/kg/dose twice daily	SR, HR control, n = 8	Amiodarone, n = 1	Bradycardia
	Kumar et al. [22]	N = 2; 1 M, 1 F	Amiodarone, esmolol, n = 1	0.1 mg/kg/d	SR, n = 2	It is not clear whether ivabradine was used as a single or adjunctive treatment	N/S
	Kumar * et al. [23]	N = 5; 3 M, 2 F	Amiodaron, esmolol, n = 5	0.1–0.2 mg/kg/d twice daily	SR, HR control, n = 5	Amiodarone, n = 2	None
	Sahu et al. [24]	N = 1; F	Magnesium sulfate, digoxin, amiodaron	0.05 mg/kg twice daily	SR, HR control	None	None
	Sharma et al. [25]	N = 4; 2 M, 2 F	Magnesium, n = 4	0.1–0.2 mg/kg/dose	SR, HR control, n = 4	None	Bradycardia

F: female; M: male; SR: sinus rhythm; HR: heart rate; TN: nodal tachycardia; JR: junctional rhythm; N/S: not specified; JET: junctional ectopic tachycardia. * Twenty patients had postoperative JET. Among these, five infants, aged seven to twelve months, had refractory JET and were treated with ivabradine.

## Data Availability

No additional data sets are associated with this paper.
